# Neuroimaging in Non-fatal Strangulation: a Retrospective Analysis of Injury Patterns, Clinical Features and Diagnostic Utility

**DOI:** 10.1007/s00062-025-01597-2

**Published:** 2025-12-09

**Authors:** Katja Döring, Zaid Abdel-Muhdy, Athanasia Warnecke, Michael Klintschar, Heinrich Lanfermann, Stefan Bleich, Johanna Seifert

**Affiliations:** 1 Institute for Diagnostic and Interventional Neuroradiology, Hannover Medical School, Carl-Neuberg-Str. 1, 30625 Hannover, Germany; 2 Department of Otolaryngology Head and Neck Surgery, Hannover Medical School, 30625 Hanover, Germany; 3 Institute of Legal Medicine, Medical University Hannover, Carl-Neuberg-Str. 1, 30625 Hannover, Germany; 4 Department of Psychiatry, Social Psychiatry and Psychotherapy, Hannover Medical School, Carl-Neuberg-Str. 1, 30625 Hannover, Germany

**Keywords:** Internal Carotid Artery Dissection, Computed Tomography Angiography, Magnetic Resonance Imaging, Suicide Attempt, Self Injurious Behavior, Evidence-Based Emergency Medicine

## Abstract

**Purpose:**

To assess the incidence and patterns of injuries after non-fatal self-inflicted (SIS) and non-self-inflicted strangulation (NSIS) and evaluate diagnostic utility of computed tomography (CT) and magnetic resonance imaging (MRI).

**Methods:**

Single-center retrospective analysis (2013–2024) of patients undergoing CT and/or MRI following SIS/NSIS. Demographics, psychiatric comorbidity, strangulation mechanism, clinical symptoms and imaging findings were analyzed. Imaging was assessed for strangulation-associated injuries (e.g., fractures of the hyoid-larynx-complex [HLC], soft tissue hematoma [STH], blunt cervicovascular injury [BCVI]). Descriptive statistics were performed to detect risk factors for strangulation-associated injuries.

**Results:**

106 patients (55.7% female; mean age 40.2 years) and 124 events of strangulation with subsequent neuroimaging were included (CT: 96.8%, MRI: 3.2%). SIS comprised 80.6% of cases, mostly ligature strangulation (68.0%), followed by near-hanging (29.0%). NSIS accounted for 18.5% of cases. Eleven patients sustained strangulation-associated injuries, primarily HLC fractures and STH (6 cases each), but no BCVI. Older age (odds ratio: 1.04; 95% confidence interval: 1.01–1.07; *p* = 0.021) showed a weak association and male sex (6.32, 1.31–30.59, *p* = 0.022), near-hanging (12.36, 3.19–47.81, *p* < 0.001) and intubation (8.65, 2.04–36.78, *p* < 0.001) a moderate association with strangulation-associated injuries. We identified a distinct patient subgroup with recurrent SIS, characterized by predominant female sex, younger age and psychiatric disorders presenting with emotional instability. Injuries were not detected in any of these cases.

**Conclusion:**

Severe strangulation-associated injuries are rare. CT should be used selectively, particularly in alert patients lacking relevant clinical findings. MRI may be preferable in younger patients and for forensic evaluation, particularly in NSIS.

**Key Points**

1. There is a distinct subset of patients characterized by mental disorders with emotional dysregulation, younger age and female sex who repeatedly obtain neuroimaging after self-inflicted strangulation.
2. Severe injuries after non-fatal strangulation are rare; fractures of the hyoid-larynx complex and soft tisse hematomas are the most common finding.

## Introduction

Suicide statistics are an important health indicator for any country. The World Health Organization estimates that more than 720,000 people worldwide die by suicide every year [1]. In 2023, more than 10,000 people in Germany died as a result of suicide, almost three times as many as fatalities in road traffic accidents [2]. In the same year, suicide by hanging, strangulation and asphyxiation was documented as the most common method of suicide in Germany, accounting for 41% of suicides [3]. The umbrella term “strangulation” can be characterized into three types: hanging, ligature strangulation and manual strangulation (throttling). In hanging, the neck is compressed by the body’s own weight using a strangulation device attached to a fixed point. Ligature strangulation is the compression of the throat by a device tightened by a force other than body weight, usually through active manual, force. Manual strangulation refers to the compression of the throat with the hands, forearm or other body parts [4]. A further distinction can be made between one-handed and two-handed chokeholds, which can produce different patterns of laryngeal and vascular compression [5].

While the aforementioned statistic indicating “strangulation” as Germany’s most common suicide method is alarming [3], it does not allow for differentiation between the three different types of strangulation. In fact, while hanging is one of the most lethal suicide methods with a mortality rate of more than 75%, second only to firearms [6], completed suicides by ligature or manual strangulation are considered rare events or even infeasible [7, 8]. This is in contrast to manual or ligature strangulation as a cause of homicide, which appear to be a comparatively common method [8].

In addition to non-self-inflicted strangulation (NSIS) and strangulation in suicidal intent, self-inflicted strangulation (SIS) is a relevant form of self-harm, alongside other methods, including cutting, burning and poisoning. These behaviors are particularly prevalent in individuals with psychiatric disorders characterized by emotional instability, such as borderline personality disorder (BPD) and post-traumatic stress disorder (PTSD). They often occur in a highly agitated state [9, 10], which can complicate further diagnostic assessment and clinical management. Moreover, these procedures may pose an additional risk to the patient (e.g., absconding if transferred from the secure ward for imaging).

Although secondary prevention and diagnosis of secondary injuries after events of hanging or strangulation are of paramount importance, data on the methods and circumstances of non-fatal strangulation attempts remain scarce. In particular, for cases of ligature or manual strangulation, data on the incidence and type of injury to laryngeal structures and cranial artery dissection are lacking. Several studies have investigated the incidence of these complications following events of near-hanging [11–14], near-hanging or manual strangulation [15] or non-specified strangulation or near-hanging [16]. Others have focused on victims of NSIS [17–22]. As this is a relevant forensic dilemma, some research has focused on the differentiation of post-mortem injuries in suicide and homicide victims [7, 8, 23–25].

Overall, studies systematically investigating the sequelae specifically of events of *self-inflicted* manual and/or ligature strangulation appear to be lacking. This gap exists despite the clinical relevance of such cases. The aim of our retrospective study is to evaluate the routine use of emergency neuroimaging at a university hospital in Germany after any type of non-fatal strangulation (SIS and NSIS; near-hanging, manual and ligature strangulation). We will focus on (a) the different methods of strangulation, (b) the diagnostic process and (c) the incidence of dissection and other neck injuries. Based on the results of this study, will we propose an optimization of the diagnostic process after strangulation. Secondary objectives are to promote a rational use of human, financial and technical resources.

## Material and Methods

This retrospective longitudinal study included all patients who received neuroimaging after SIS or NSIS at Hannover Medical School between January 2013 and December 2024. For each case, the following information was retrieved from the electronic medical record and the image archive: age, sex, mechanism of strangulation (i.e., hanging, manual, ligature or undocumented), context of strangulation (self-harm/suicide attempt, domestic violence, unknown assailant, accidental or undocumented), type of imaging performed (computed tomography [CT], computed tomography angiography [CTA], polytrauma CT, magnetic resonance imaging [MRI], magnetic resonance angiography [MRA]) and clinical and imaging findings. As noted in the introduction, events of strangulation are classified as “hanging”, “manual strangulation”/“throttling” or “ligature strangulation” (according to [4]). Due to the retrospective nature of the present study, our ability to assign precise categories was constrained by the quality of existing documentation. Where records permitted, we specified the type of strangulation. The umbrella term “strangulation” refers to any type of event.

The imaging examinations were independently assessed by two neuroradiologists for the presence of soft tissue hematoma (STH), fractures of the hyoid-larynx complex (HLC) and/or injuries to the cervical spine, blunt cervical vascular injury (BCVI) and hypoxic brain injury (HBI). Other secondary injuries (e.g., limb fractures) were recorded separately. The injuries were classified as ‘injuries directly associated with strangulation’, including the following injury patterns: STH, HLC, BCVI, HBI and discoligamentous injuries, and ‘secondary injuries not directly associated with strangulation’, including all other injuries. This retrospective classification was used as a reference standard for evaluating CTA of the head and neck vessels, native CT of the neck organs, and MRI scans of the neck organs or MR angiography of the head and neck vessels. In cases in which CT polytrauma was used, the same classification was applied based on the available imaging data. Additional thoracoabdominal findings and fractures of the rest of the skeleton were recorded separately in these cases.

### Statistical Analysis

Statistical analysis was performed using SPSS, Version 29.0. The level of significance was set at *p* < 0.05. Descriptive statistics were used to analyze the study population, the type and quality of neuroimaging, imaging findings, patient status and clinical symptoms. Continuous variables, such as patient age, are expressed as means, medians and ranges. Categorical variables and qualitative parameters, such as patient sex, mechanism of strangulation, context of strangulation, type of imaging study performed and number of injuries reported, are expressed as total count (*n*) and percentages. Means and standard deviation (SD) were calculated for metric variables (i.e., age).

In order to identify risk factors for injuries following NSIS and SIS, we chose to only include variables that can easily and objectively be assessed in the emergency setting (e.g., age, sex, intubation), while variables such as psychiatric comorbidity were excluded, as these may not be readily available or reliable. Univariate analysis was performed using the Mann-Whitney U test for age and Fisher’s exact test for categorical variables (e.g., sex, method used, sedation). Subsequently, correlation analyses were performed to determine the significance of the association between strangulation-associated injuries and the variables which yielded significant results in univariate analyses. The Eta coefficient was used for the metric variable age and the Phi coefficient was used for categorical variables. Effect sizes were interpreted as no correlation (0), weak (0.1), moderate (0.3), strong (0.5) and perfect (1.0). Due to the small sample size (*n* = 124) and the low number of injuries (*n* = 11), a further multivariate logistic regression analysis was not feasible.

## Results

### Study Population

**Fig. 1:**
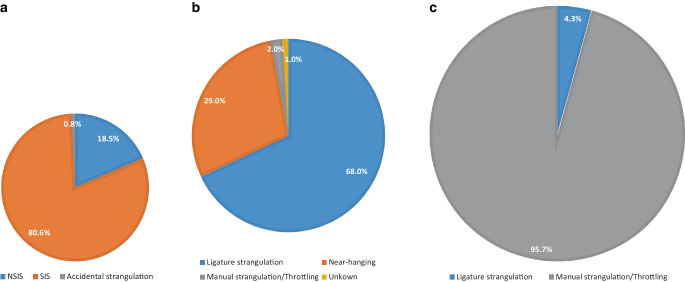
**a** Proportion of cases of self-inflicted strangulation (SIS; *n* = 100) and non-self-inflicted strangulation (NSIS; *n* = 23). One accidental strangulation was recorded (0.8%). **b** Type of strangulation in self-inflicted strangulation (SIS; *n* = 100). In the majority of SIS cases, ligature strangulation (*n* = 68) was the most common strangulation method, followed by near-hanging (*n* = 29) and manual strangulation combined with suffocation (e.g., with a plastic bag, *n* = 2). In one case, the type of strangulation could not be determined from the patient’s medical history. **c** Manual strangulation/throttling was the predominant mechanism in cases of NSIS (95.6% vs. ligature strangulation 4.3%), most often due to intimate partner violence in 96% of cases (*n* = 23; 22 females, 1 male)

A total of 124 events of strangulation with subsequent neuroimaging studies in 106 patients following SIS and NSIS were performed during the nearly 11-year study period. The mean age of patients was 40.2 years (SD 19.3), 55.7% were female (Table 1). The majority of cases were SIS (100 cases, 80.6% vs. NSIS in 23 cases, 18.5%). One accidental strangulation was recorded (0.8%; Fig. 1a). In the majority of the 100 SIS cases, ligature strangulation (*n* = 68, 68.0%) was performed, followed by near-hanging (*n* = 29, 29.0%) and strangulation combined with suffocation (e.g., with a plastic bag, *n* = 2, 2.0%; Fig. 1b). Manual strangulation/throttling was the predominant mechanism in cases of NSIS (95.7%; Fig. 1c). NSIS was most often due to intimate partner violence in 96.0% of cases (*n* = 23; 22 females, 1 male). Assault by an unknown perpetrator accounted for 4.0% of cases (*n* = 1; 1 male).

**Table 1:** Baseline characteristics

	Count	Percent (%)	Mean, SD, range
**Total number of patients**	106	100	–
**Sex**			
Male	47	44.3	–
Female	59	55.7	–
**Age (in years)**	–	–	40.2, 19.3, 8–98
**Total number of scans**	124	100	–
**Injuries detected in imaging (n** **=** **124)**			
*No injuries*	109	87.9	–
Injuries directly associated with strangulation*	11	8.9	–
STH	6	4.8	–
Dens fracture	2	1.6	–
HLC fracture	6	4.8	–
Discoligamentous injury	2	1.6	–
BCVI	0	0.0	–
HBI	0	0.0	–
Secondary injuries not directly associated with strangulation*	9	7.3	–
Midface fracture	4	3.2	–
Limb fracture	3	2.4	–
Stability-compromising vertebral fracture	2	1.6	–
Non-stability-compromising vertebral fracture	2	1.6	–
Rib series fracture	3	2.4	–
Stable pelvic fracture	1	0.8	–
Sternum fracture	1	0.8	–
ICH	2	1.6	–
**Imaging modality (*n*** **=** **124)**			
Polytrauma CT	7	5.6	–
CT of the neck	20	16.1	–
CTA of the head and neck vessels	93	75.0	–
MRI of the neck	4	3.2	–
**Quality of imaging (*n*** **=** **124)**			
Good	89	71.8	–
Reduced	27	21.8	–
Poor	8	6.5	–
**Additional measures required (*n*** **=** **124)**			
None	77	62.1	–
Sedation	17	13.7	–
Mechanical restraint	21	16.9	–
Intubation	9	7.3	–

*SD* standard deviation; *CT* computed tomography; *CTA* computed tomography angiography; *MRI* magnetic resonance imaging; *STH* soft tissue hematoma; *BCVI* blunt cervicovascular injury; *HLC* hyoid-larynx-complex; *HBI* hypoxic brain injury; *ICH* intracranial hemorrhage

*patients may have contracted more than one injury, see also Table 2

**Fig. 2:**
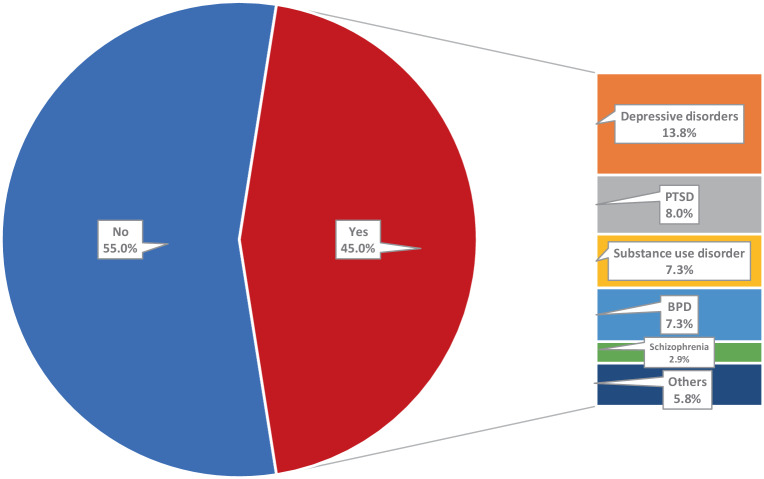
Prevalence and distribution of psychiatric disorders. *BPD* borderline personality disorder, *PTSD* posttraumatic stress disorder

Psychiatric comorbidities were documented in 45.0% (*n* = 45) of the 100 SIS cases, with depressive disorders (13.8%) and PTSD (8.0%) being the most common diagnoses (Fig. 2). Eleven patients (10.4% of 106 patients) presented with numerous attempts of SIS within the study period. These patients had an average age of 31 years (SD 9.4), were predominantly female (*n* = 8; 72.7%) and represented ¼ of the total neuroimaging studies. Within this patient subgroup, an average of 2.6 (SD 1.56) scans were performed per person within the study period. The most common psychiatric diagnoses in this patient group were BPD (54%) and PTSD (27%). One patient (female, 31 years, BPD) underwent 11 CCT/CTA of the head and neck vessels within one year. Another patient (male, 29 years, PTSD) underwent eight CCT/CTA of the head and neck vessels and two MRA of the head and neck vessels within a two-year period. There was no evidence of pre-existing psychiatric illness in any of the 23 cases of NSIS (data not shown in tables or figures).

### Imaging Modality, Quality and Additional Measures Required

**Fig. 3:**
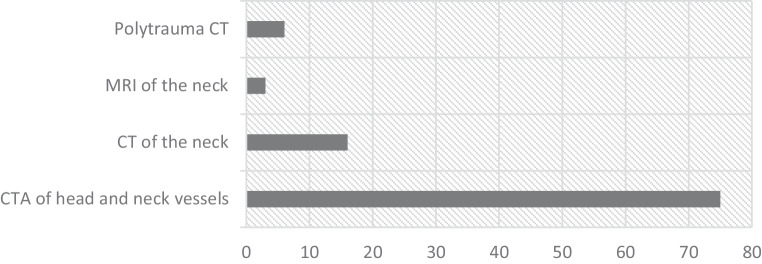
Overview of imaging modalities used [in percent]. Depending on the severity of the injuries, the vigilance of the patient or unclear supine trauma, an interdisciplinary decision was made on the imaging modality required. *CT(A)* computed tomography (angiography), *MRI* magnetic resonance imaging

A total of 124 imaging studies were performed (CCT/CTA: *n* = 120, 96.8%; MRI: *n* = 4, 3.2%). Most commonly, CTA of the head and neck vessels (*n* = 93, 75.0% of CTs) was performed followed by native CT of the neck (*n* = 20, 16.1% of CTs; Table 1; Fig. 3). Almost one third (*n* = 35, 28.2%) of the 124 imaging studies performed were of reduced (*n* = 27, 21.8%) or poor (*n* = 8, 6.5%) quality (Table 1). In four cases, despite use of mechanical restraint, the CT scans were so artefact-laden that they could not be properly evaluated.

Polytrauma CTs were performed on seven clinically severely affected individuals (5.6% of the imaging studies), all of which were SIS. In four of these seven cases (2 females, 2 males), near-hanging had been performed with a subsequent fall from great height. In 7.3% (*n* = 9) of all neuroimaging studies, patients had been intubated prior to imaging, while 17 patients (13.7%) required sedation. Mechanical restraint of the patient was required in 21 cases (16.9%; Table 1).

### Symptoms Detected During Physical Examination

In 57 cases (46.0% of 124), the patients reported otorhinolaryngological symptoms during physical examination. The following symptoms were reported in descending order: Neck pain (*n* = 22, 17.7%), dysphagia (*n* = 18, 14.5%), vocal cord edema/pharyngeal swelling (*n* = 8, 6.5%) and dyspnea (*n* = 4, 3.2%). Neurological deficits were observed in four patients (3.2%). Visible strangulation marks were found in less than one third (*n* = 37, 29.8%) of cases. The following external injuries were observed in increasing frequency: Edema (*n* = 1, 0.8%), excoriation (*n* = 2, 1.6%), petechiae (*n* = 4, 3.2%) and erythema (*n* = 7, 5.6%; data not shown in tables or figures).

### Injuries Visible in Neuroimaging After Strangulation

In 109 cases, there were no relevant imaging findings after either SIS or NSIS (no injury: 109/124, 87.9%), while visible injuries were present in 15 cases (12.1%). Eleven patients sustained injuries directly associated with strangulation (8.9%; see Tables 1 and 2). Three patients were victims of NSIS via manual strangulation, in so, the incidence of strangulation-associated injuries in NSIS (*n* = 23) was 13.0%. Six patients had survived an incident of near-hanging (*n* = 29), corresponding to an incidence of injuries of 20.7%. The remaining two patients had performed ligature strangulation on themselves, indicating an incidence of injuries of 2.9% of this method (*n* = 68; compare also with Table 2 and Fig. 1a–c). A majority of patients with strangulation-associated injuries were male (*n* = 9) with an average age of 47.7 years (SD 12.2). Psychiatric comorbidities were documented in nine cases, most commonly depressive disorder. In five cases, the patients had been sedated and intubated prior to imaging (Table 2).

**Table 2:** Detailed list of patients who sustained injuries directly associated with strangulation.

No.	SIS	NSIS	Age	Imaging modality	Sex	Psychiatric disorder	Near-hanging	Manual/ligature strangulation	Tool	ENT symptoms	Measures required	Radiological findings
1	X	–	59	Polytrauma CT	male	Depressive disorder	X	–	Rope	Swelling	sedated, intubated	HLC fracture
2	X		49	CTA	male	Depressive Disorder	X		Unknown	Strangulation marks		HLC fracture
												STH
3	X		72	CTA	female	Alcohol abuse		X	Belt	Neck pain		HLC fracture
4	X		48	CTA	male	Depressive disorder		X	Belt	Neck pain, dysphonia, dysphagia		HLC fracture
												STH
5		X	48	CTA	male			X	Hands			HLC fracture
6	X		34	MRA, CT	male	Depressive disorder	X		Rope	Strangulation marks		HLC fracture
7	X		40	Polytrauma CT	male		X		Rope		sedated, intubated	Dens axis fracture
												vertebral fracture
												compromising stability of L3 and L4
												Limb fracture
												Midface fracture
												STH
8	X		44	Polytrauma CT	male	Depressive disorder	X		Rope		sedated, intubated	Dens axis fracture
												Discoligamentous injury of C6/C7
												Epidural haematoma on the right parietal
												Bitemporal ICH
9	X		58	Polytrauma CT	male	Depressive disorder	X		Rope		sedated, intubated	Midface fracture
												STH
10		X	45	CTA	female	–	–	X		Neck pain		Midface fracture
												STH
11		X	28	CTA	male	Post-traumatic stress disorder		X	Hands	Neck pain, dysphonia, dysphagia		Midface fracture
												rib series fracture
												STH

*(N)SIS* (non-)self-inflicted strangulation; *CT* computed tomography; *CTA* computed tomography angiography of head and neck vessels; *MRA* magnetic resonance angiography of head and neck vessels; *ENT* ear, nose, throat; *HLC* hyoid-Larynx complex fracture; *HBI* hypoxic brain injury; *ICH* intracranial hemorrhage

The most common types of strangulation-associated injuries were HLC fractures and STH (6 cases each), followed by injuries of the cervical spine were discoligamentous injuries (2 cases) and dens fractures (2 cases). Secondary injuries were only detected in nine patients (Table 2), of which five had also sustained strangulation-associated injuries. The following additional secondary strangulation-associated injuries were identified in the imaging examinations (in descending order of frequency): Midface fractures (*n* = 4), limb fractures (*n* = 3), rib series fractures (*n* = 3), stability-compromising vertebral fractures (*n* = 2), non-stability-compromising vertebral fractures (*n* = 2), intracranial hemorrhages (ICH; *n* = 2), stable pelvic fractures (*n* = 1) and sternum fractures (*n* = 1; Table 1).

### Risk Factors for Injuries Directly Associated with Strangulation

In the univariate analysis, the occurrence of injuries directly associated with strangulation significantly correlated with increasing age (OR 1.04, CI 1.01–1.07, *p* = 0.021), male sex (OR 6.32, CI 1.31–30.59, *p* = 0.022), hanging (OR 12.36, OR 3.19–47.81, *p* *<* 0.001) and intubation (OR 8.65, CI 2.04–36.78, *p* = 0.008), but not for mechanical restraint or any of the clinical findings. Correlation analysis revealed a weak association with age (Eta = 0.123, Eta^2^ = 0.015) and moderate correlations with sex (Phi = 0.230), hanging as the method used (Phi = 0.388) and intubation (Phi = 0.302; Table 3).

**Table 3:** Risk factors for injuries directly associated with strangulation.

Variable (reference)	Test used	Test statistics	*p*-value	Odds ratio (95% confidence interval)	Effect size
Age in years	Mann-Whitney U	Mann-Whitney	0.021*	1.04 (1.01–1.07)	Eta = 0.123; Eta^2^ = 0.015
		U = 391.0			
		Z‑statistic = −2.027			
Sex (male)	Fischer’s Exact	–	0.022*	6.32 (1.31–30.59)	Phi = 0.229
Method used (hanging)	Fischer’s Exact	–	0.001*	12.36 (3.19–47.81)	Phi = 0.388
Intubation (yes)	Fischer’s Exact	–	0.008*	8.65 (2.04–36.78)	Phi = 0.302
Sedation (yes)	Fischer’s Exact	–	0.065	–	–
Mechanical restraint (yes)	Fischer’s Exact	–	1.0	–	–
Neck pain (yes)	Fischer’s Exact	–	0.410	–	–
Dyspnea (yes)	Fischer’s Exact	–	1.0	–	–
Dysphonia (yes)	Fischer’s Exact	–	0.377	–	–
Dysphagia (yes)	Fischer’s Exact	–	1.0	–	–
SIS vs NSIS (SIS)	Fischer’s Exact	–	1.0	–	–

*SIS* self-inflicted strangulation; *NSIS* non-self-inflicted strangulation

## Discussion

The present study assessed findings from a total of 124 neuroimaging studies in patients following both self-inflicted and non-self-inflicted strangulation focusing on secondary injuries directly associated with strangulation. Neuroimaging yielded strangulation-associated injuries in only eleven cases, most commonly STH and HLC fractures, but no BCVI. Risk factors for strangulation-associated injuries were older age, male sex, near-hanging and previous intubation. We further identified a distinct subgroup of patients with recurrent events of SIS and subsequent neuroimaging, who were mostly younger females suffering from mental health disorders characterized by emotional dysregulation.

Injuries directly associated with strangulation were rare and mainly comprised HLC fractures and STH. The risk factors detected (i.e., older age, male sex, near-hanging) are consistent with well-known demographic risk factors for completed suicide and reflect the greater force applied in this highly lethal suicide method [26, 27]. On the other hand, completed suicide following ligature or manual strangulation performed on one’s self has been considered infeasible by several experts [7, 8]. Compression of the carotid arteries generally leads to a rapid loss of consciousness as a result of decreased cerebral blood flow. Once unconscious, it is physiologically impossible for an individual to maintain sufficient pressure on the ligature, in turn leading to its release and restoration of blood flow [4]. In a systematic review, Cordner et al. summarized a total of 31 case reports of suicidal ligature strangulation and found that the victims had implemented means by which pressure would be upheld after loss of consciousness, such as through knotting or attachment to objects to assist with compression [28]. However, one could argue that this goes beyond a ligature strangulation per se and perhaps more closely resembles (atypical) hanging.

To the best of the authors’ knowledge, studies systematically examining the sequelae in survivors of SIS via ligature or manual strangulation are not available, indicating that injuries are either rare or under-detected. This makes the two cases of HLC fractures following SIS particularly remarkable. Both patients had used belts and presented with additional ENT symptoms. However, compared to near-hanging and NSIS, which both had a higher incidence of strangulation-associated injuries of 20.7% and 13.0%, injuries following SIS via ligature strangulation were rare (2.9%). Other researchers previously concluded that not only completed suicide [7, 8], but also fractures following self-inflicted ligature or manual strangulation are highly improbable, arguing that the onset of pain, airway obstruction or respiratory compromise would cause the individual to rapidly release the constricting force, thus preventing the sustained pressure required to induce such an injury. In fact, the same authors point out, that the presence of fractures following strangulation should be considered indicators of third-party involvement [29]. Fractures following NSIS are a frequent finding in up to 71% of victims, especially in fatal cases [30–32]. In survivors of strangulation, a majority of fractures present with additional clinical features, mainly dyspnea, stridor, dysphonia and cervical pain. These symptoms may be discrete or of delayed onset, generally presenting within 48 hours [29]. While fractures following manual/ligature autostrangulation appear rare, fractures after near-hanging are the most common type of injury sustained after near-hangings. Ribaute et al. reported a similar incidence of 25% in near-hanging victims treated in the intensive care unit, but also in 13% of patients with more mild symptoms (i.e., hemodynamic stability and preserved consciousness) [14].

The present study was unable to detect even a single account of BCVI. In a retrospective cross-sectional study from 2023 in a psychiatric setting, Etgen et al. detected two cases of dissection in a total of 99 patients with SIS of which 50 patients underwent neuroimaging. In one case, an older man had attempted to hang himself, while in the other the strangulation method was not disclosed, though it was mentioned that the patient had presented with mild anisocoria [16]. Also aiming to determine the incidence of BCVI, Ribaute et al. retrospectively examined CTAs of 162 patients after nonfatal near-hanging. They detected a total of four dissections (three vertebral and one external carotid artery), all of which were found in male patients presenting with cardiac arrest upon arrival to the emergency room. Under consideration of this finding, the authors determined that CTAs may only be appropriate in the most severely affected patients [14].

Apart from older age, male sex and near-hanging, intubation significantly predicted strangulation-associated injuries. Intubation indirectly indicates a more critical condition and a lower Glasgow Coma Scale (GCS) score of the affected patient (i.e., those requiring airway management or with traumatic brain injury) and is generally performed using sedation [33]. Previous studies have been able to include GCS scores, indicating that lower scores relevantly coincide with the incidence of post-strangulation injuries [11, 14, 15]. Matusz et al. specifically examined injuries after nonfatal strangulation in 349 alert patients (i.e., patients with a GCS score of ≥ 13) including 21 near-hangings and 328 manual strangulations. Unfortunately, they did not further disclose, whether the events of manual strangulations were self-inflicted or not. They detected injuries in six patients (one near-hanging, five NSIS), all of which complained of abnormal symptom or presented with abnormal findings. Two female victims of NSIS suffered from cervical artery dissection, which were the only two injuries deemed clinically relevant by the authors [15]. Subramanian et al. examined 125 patients after near-hanging and categorized patients according to their GCS (normal = 15, abnormal < 15). Abnormal GCS was classified as mild (13–14), moderate (8–12) or severe (3–8). Eighty-three patients had a GCS of 15 of which 68 underwent imaging. Injuries were detected in two patients (one C5 fracture, one vertebral artery injury), both of whom also reported additional symptoms (e.g., cervical tenderness, dysphonia, dysphagia). All patients presenting with a GCS of < 15 underwent imaging. No injuries were detected in any of the seven patients with a mildly abnormal GCS, while those with moderately and severely reduced scores suffered from increasing injuries in a linear relationship. These findings lead the authors to conclude that imaging should be not be routinely performed in alert patients in the absence of other signs and symptoms of trauma. Rather, these patients should primarily be clinically monitored before further diagnostic measures are taken [11].

A careful assessment of clinical findings and alertness appears particularly relevant within the subset of patients with recurrent events of SIS detected within this study, none of which sustained strangulation-associated injuries. The demographic characteristics of this subgroup (i.e., young females with mental health disorders characterized by emotional dysregulation) are indicative of strangulation as a form of non-suicidal self-injury (NSSI) [34, 35]. NSSI generally serves the purpose of tension relief or regulation of intense emotions without the primary intent of completed suicide [36], suggesting that patients apply lesser strangulation force, corresponding to a low risk of subsequent injury. Emotional dysregulation in these scenarios may present with extreme agitation, which in turn requires iatrogenic sedation and/or mechanical restraint as prerequisite for further diagnostic work-up, reducing the quality of imaging. These considerations suggest that emergency neuroimaging may be dispensable for this patient group under these adverse conditions, at least in the acute phase.

Given the young age (40.3 years) of our study collective, the use of ionizing radiation is a particular concern. First, younger age was not associated with detectable injuries in neuroimaging after non-fatal strangulation in the present study, especially in the subgroup of patients with recurrent strangulation (31.3 years). The average radiation dose for a carotid CTA is 4.85 mSv [37]. In so, the patient who attempted eleven strangulations in one year, as reported in the results, was exposed to 53.35 mSv. This is equivalent to the cumulative effective dose of the annual radiation exposure from artificial radiation expected over 33 years [38]. The use of CT technology is a cause for concern in terms of radiation protection, particularly among children and young adults, considering that there is a significant dose-response relationship between CT radiation exposure and brain cancer [39]. This alarming finding should serve as a reminder to always carefully consider the (long-term) costs of the use of potentially damaging ionizing radiation.

When compared with previous studies [11–16] with similar aims to our own, there is a great deal of heterogeneity: The numerous differences between these studies include the type of study center (emergency department, psychiatric hospital), heterogeneous inclusion and exclusion criteria (especially regarding the type of strangulation), the different use of imaging techniques (CT/MRA, ultrasonography) and sample size (ranging from 71 to 349 patients). This explains the different results and conflicting recommendations for further diagnostics to detect secondary injuries after nonfatal strangulation. Whereas some studies question the use of systematic neuroimaging [11, 13–15], others tend to support its use [12] or refrain from making any specific recommendations [16]. On the one hand, CTA carries a risk of adverse reactions to contrast media and uses potentially harmful x‑rays, both of which significantly limit its suitability as a screening method. On the other hand, the oversight of a secondary injury after a non-fatal strangulation, such as a carotid artery dissection, carries the risk of a permanent disability or fatal stroke.

The authors of the present study acknowledge the benefits of neuroimaging in the detection of injuries following strangulation. However, the fact that we were unable to detect even a single event of carotid artery dissection raises the question, whether CTA should be performed as a standard procedure after strangulation.

To date, there are no diagnostic and documentation standards for imaging in strangulation victims. Studies using MRI in both living and deceased strangulation victims have revealed that victims have several distinct but often discrete findings, particularly in the soft tissues of the neck. The injuries are indicative of a previous strangulation injury but need to be reliably sought and recognized by the examiner. These findings may be crucial for the victim in any subsequent legal proceedings [7, 40]. The sensitivity and specificity of MRI for the diagnosis of internal carotid artery (ICA) dissection range from 84% to 99% in the literature [41]. Intramural hematoma is often visible early on, in particular an increase in parietal hematoma volume in the majority of patients during the first two weeks and an increase in the degree of ICA stenosis in half of the patients [42]. CTA offers higher spatial resolution than MRI for assessing the luminal caliber of severely stenotic vessels [43]. In addition, the radiation exposure associated with CTA must be considered in the discussion of the imaging modality of choice for the initial diagnosis and follow-up assessment of ICA dissection [44]. In a comparison of MRI and CT, Debette et al. concluded that MRI is the method of choice for identifying mural hematomas in vascular dissections. Assessment of the vascular lumen (stenosis severity, occlusion, pseudoaneurysm) can be performed using CTA or MR angiography, with digital subtraction angiography as the gold standard when non-invasive imaging is inconclusive or an intervention is required. Both CT and MRI have advantages and pitfalls that limit their sensitivity and specificity [45]. MRI is the method of choice for assessing intracranial complications such as ischemia [46] and shearing injuries [47]. Color duplex sonography can be used to visualize mural vascular hematomas in dissections as a thickened hypoechoic wall. Most ICA dissections originate near the carotid bulb, a region easily accessible to ultrasonography. Previous studies report that color-coded duplex sonography detects ICA dissection with a sensitivity of 71% in clinically asymptomatic patients and up to 95% in patients with clinical cerebral ischemia [41].

**Fig. 4:**
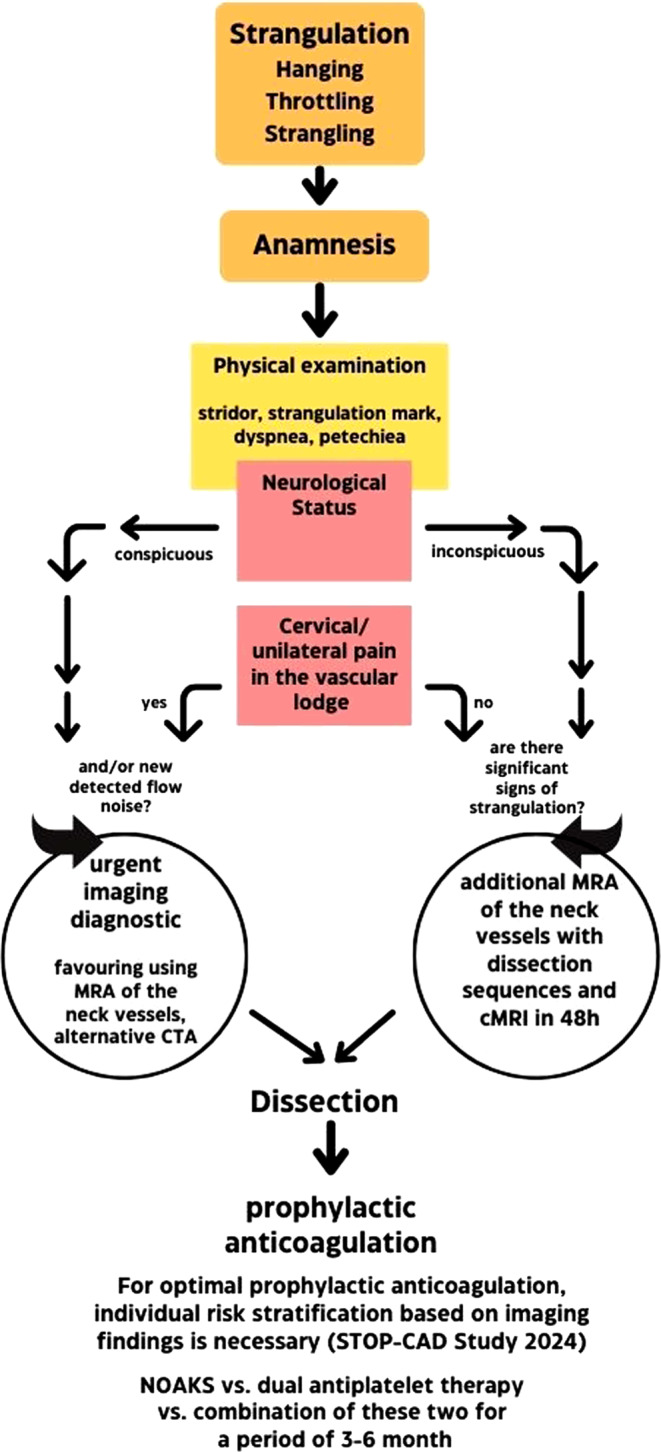
Recommended procedure for non-fatal strangulation

It can be concluded that patients presenting after strangulation with abnormal neurological status, cervical or unilateral pain or a new flow murmur should undergo urgent vascular imaging. In the absence of any of the above findings, but with clear signs of strangulation imaging within 48 hours should be considered. In these cases, MRI of the skull and soft tissues of the neck including MR angiography and dedicated dissection sequences should be prioritized. In the presence of NSIS, the case should be discussed with a forensic pathologist, as clinically asymptomatic soft tissue, vascular and even intracerebral injuries can be present (and detectable) even in the acute phase. Although CT is usually the most readily available imaging technique in emergencies and is sufficient in detecting vascular injury, it does not replace an MRI at a later stage. Especially in cases of domestic violence with potential repeated assaults, comprehensive imaging documentation obtained at a later time may be critical during legal proceedings. We have therefore developed a flowchart outlining the recommended procedure (see Fig. 4).

## Limitations

Limitations of our study include the single-center design. The monocentric nature and the narrow inclusion criteria of the study results in the relatively small number of cases. Hanging is also a suicide method with a high mortality rate; therefore, the present study only includes a small percentage of survivors of this suicide method who were consecutively presented in the emergency room. Patients who did not receive neuroimaging post-strangulation were also not included. This selection bias may potentially skew the data to include more severe cases (e.g., patients who were clinically more severely affected or presented with certain symptoms) or a certain type of patients (e.g., patients with artificial disorders who intentionally produce symptoms to receive medical attention). Due to its unavailability, the present study is also missing relevant clinical data, such as the GCS score, which is a key indicator of vigilance and neurological function. Psychiatric comorbidities were also most likely largely under-reported and not systematically assessed. Moreover, the overall low sample size of patients with strangulation-associated injuries after non-fatal strangulation reduces the statistical power of the statistical analyses and contributes to wide 95% CI.

## Conclusion and Clinical Implications

Injuries directly associated with strangulation after non-fatal strangulation, in particular dissection of cervical vessels, are rare. Other injuries, especially fractures of the HLC, are more common, but appear to mainly occur in severely affected patients after incidents of near-hanging and in patients with reduced consciousness. Neuroimaging, especially CT and CTA, are commonly used for acute evaluation of injuries in patients after non-fatal strangulation. However, its routine use in alert patients without neurological deficits or clinical signs of trauma should be reconsidered due to the reduced diagnostic utility and unnecessary radiation exposure. MRI, on the other hand, has a superior sensitivity for soft tissue and vascular injuries, and should be prioritized within a 48-hour timeframe in forensic cases, especially NSIS, in which sufficient legal documentation is essential.

A thorough clinical evaluation, including the assessment of the mechanism of strangulation (self-inflicted vs. non-self-inflicted, use of hands vs. use of tools, near-hanging vs. ligature/manual strangulation), remains critical in guiding the rational use of imaging in patients after non-fatal strangulation. While patients with neurological deficits, reduced consciousness, cervical pain or other abnormal physical findings require urgent imaging, a more conservative approach may be sufficient in asymptomatic patients. Neuroimaging in patients that require mechanical restraint due to severe agitation should be performed with caution. Restricting imaging to cases with clear clinical indications can optimize resource allocation and prevent unnecessary procedures and radiation exposure. Further systematic assessments on strangulation outcomes are needed to guide evidence-based risk assessment and clinical decision-making in these events to ensure both patient safety and judicial integrity. Future studies should enroll larger samples and systematically collect variables such as the GCS, vital signs and a more detailed psychiatric history to clarify the relationship between injury characteristics, clinical status and mental health history in patients following non-fatal strangulation.

## Abbreviations

BCVI: Blunt cervicovascular injury
BPD: Borderline personality disorder
CI: Confidence interval
CT: Computed tomography
CTA: Computed tomography angiography
HBI: Hypoxic brain injury
HLC: Hyoid-larynx-complex
ICA: Internal carotid artery
ICH: Intracranial hemorrhage
MRA: Magnetic resonance angiography
MRI: Magnetic resonance imaging
NSIS: Non-self-inflicted strangulation
OR: Odds ratio
PTSD: Post-traumatic stress disorder
SD: Standard deviation
SIS: Self-inflicted strangulation
STH: Soft tissue hematoma

## References

[1] World Health Organization: Suicide. 2025. https://www.who.int/news-room/fact-sheets/detail/suicide. Accessed 28 Aug 2025.

[2] 2025. https://www.destatis.de/DE/Themen/Laender-Regionen/Internationales/Thema/bevoelkerung-arbeit-soziales/gesundheit/Suizid.html. Accessed 2028.

[3] Statistisches Bundesamt. 2017. https://de.statista.com/statistik/daten/studie/585/umfrage/selbstmordmethoden-in-deutschland-2006/. Accessed 2028.

[4] Dunn RJ, Sukhija K, Lopez RA. Strangulation injuries. 2017. 29083611

[5] Härm T, Rajs J. Types of injuries and interrelated conditions of victims and assailants in attempted and homicidal strangulation. Forensic Sci Int. 1981;18(2):101–23. 10.1016/0379-0738(81)90148-1. 7297963 10.1016/0379-0738(81)90148-1

[6] Cai Z, Junus A, Chang Q, Yip PSF. The lethality of suicide methods: A systematic review and meta-analysis. J Affect Disord. 2022;300:121–9. 10.1016/j.jad.2021.12.054. 34953923 10.1016/j.jad.2021.12.054

[7] Gascho D, Heimer J, Tappero C, Schaerli S. Relevant findings on postmortem CT and postmortem MRI in hanging, ligature strangulation and manual strangulation and their additional value compared to autopsy—a systematic review. Forensic Sci Med Pathol. 2019;15(1):84–92. 10.1007/s12024-018-0070-z. 30627977 10.1007/s12024-018-0070-z

[8] Maxeiner H, Bockholdt B. Homicidal and suicidal ligature strangulation—a comparison of the post-mortem findings. Forensic Sci Int. 2003;137(1):60–6. 10.1016/S0379-0738(03)00279-2. 14550616 10.1016/s0379-0738(03)00279-2

[9] Burckell LA, McMain S. Contrasting clients in dialectical behavior therapy for borderline personality disorder: “Marie” and “Dean,” two cases with different alliance trajectories and outcomes. Pragmat Case Stud Psychother. 2011;7(2):246–67. 10.14713/pcsp.v7i2.1090.

[10] Andover MS, Gibb BE. Non-suicidal self-injury, attempted suicide, and suicidal intent among psychiatric inpatients. Psychiatry Res. 2010;178(1):101–5. 10.1016/j.psychres.2010.03.019. 20444506 10.1016/j.psychres.2010.03.019

[11] Subramanian M, Hranjec T, Liu L, Hodgman EI, Minshall CT, Minei JP. A case for less workup in near hanging. J Trauma Acute Care Surg. 2016;81(5):925–30. 10.1097/ta.0000000000001231. 27537511 10.1097/TA.0000000000001231

[12] Schellenberg M, Inaba K, Warriner Z, Alfson D, Roman J, Van Velsen V, et al. Near hangings: Epidemiology, injuries, and investigations. J Trauma Acute Care Surg. 2019;86(3):454–7. 10.1097/ta.0000000000002134. 30444857 10.1097/TA.0000000000002134

[13] Schuberg S, Gupta N, Shah K. Aggressive imaging protocol for hanging patients yields no significant findings: Over-imaging of hanging injuries. Am J Emerg Med. 2019;37(4):737–9. 10.1016/j.ajem.2019.01.006. 30630681 10.1016/j.ajem.2019.01.006

[14] Ribaute C, Darcourt J, Patsoura S, Ferrier M, Meluchova Z, Gramada R, et al. Should CT angiography of the supra-aortic arteries be performed systematically following attempted suicide by hanging? J Neuroradiol. 2021;48(4):271–6. 10.1016/j.neurad.2019.04.001. 31034897 10.1016/j.neurad.2019.04.001

[15] Matusz EC, Schaffer JT, Bachmeier BA, Kirschner JM, Musey PI Jr., Roumpf SK, et al. Evaluation of Nonfatal Strangulation in Alert Adults. Ann Emerg Med. 2020;75(3):329–38. 10.1016/j.annemergmed.2019.07.018. 31591013 10.1016/j.annemergmed.2019.07.018

[16] Etgen T, Stigloher M, Förstl H, Zwanzger P, Rentrop M. Systematic analysis of nonfatal suicide attempts and further diagnostic of secondary injury in strangulation survivors: A retrospective cross-sectional study. Health Sci Rep. 2023;6(10):e1572. 10.1002/hsr2.1572. 37795312 10.1002/hsr2.1572 PMC10545888

[17] Shields LBE, Corey TS, Weakley-Jones B, Stewart D. Living Victims of Strangulation: A 10-Year Review of Cases in a Metropolitan Community. Am J Foren Med Pathol. 2010;31(4):320–5. 10.1097/PAF.0b013e3181d3dc02. 10.1097/paf.0b013e3181d3dc02 21171201

[18] Bauer M, Hollenstein C, Lieb JM, Grassegger S, Haas T, Egloff L, et al. Longitudinal visibility of MRI findings in living victims of strangulation. Int J Legal Med. 2024;138(4):1425–36. 10.1007/s00414-024-03207-1. 38561435 10.1007/s00414-024-03207-1 PMC11164791

[19] Bruguier C, Genet P, Zerlauth J‑B, Dédouit F, Grimm J, Meuli R, et al. Neck-MRI Experience for Investigation of Survived Strangulation Victims. Forensic Sci Res. 2019;5(2):113–8. 10.1080/20961790.2019.1592314. 32939427 10.1080/20961790.2019.1592314 PMC7476612

[20] Heimer J, Tappero C, Gascho D, Flach P, Ruder TD, Thali MJ, et al. Value of 3T craniocervical magnetic resonance imaging following nonfatal strangulation. Eur Radiol. 2019;29(7):3458–66. 10.1007/s00330-019-06033-x. 30796576 10.1007/s00330-019-06033-x

[21] Christe A, Thoeny H, Ross S, Spendlove D, Tshering D, Bolliger S, et al. Life-threatening versus non-life-threatening manual strangulation: are there appropriate criteria for MR imaging of the neck? Eur Radiol. 2009;19(8):1882–9. 10.1007/s00330-009-1353-2. 19283386 10.1007/s00330-009-1353-2

[22] Christe A, Oesterhelweg L, Ross S, Spendlove D, Bolliger S, Vock P, et al. Can MRI of the neck compete with clinical findings in assessing danger to life for survivors of manual strangulation? A statistical analysis. Leg Med. 2010;12(5):228–32. 10.1016/j.legalmed.2010.05.004. 10.1016/j.legalmed.2010.05.004 20630784

[23] Commins C, Bolster M, Mulligan L. To investigate the pattern of neck injuries and the role of toxicology in cases of hanging and manual/homicidal ligature strangulation in Ireland between 2016–2020: A retrospective review and analysis. J Forensic Leg Med. 2024; 10.1016/j.jflm.2024.102686. 38692099 10.1016/j.jflm.2024.102686

[24] Deininger-Czermak E, Heimer J, Tappero C, Thali MJ, Gascho D. Postmortem Magnetic Resonance Imaging and Postmortem Computed Tomography in Ligature and Manual Strangulation. Am J Forensic Med Pathol. 2020;41(2):97–103. 10.1097/paf.0000000000000542. 32205490 10.1097/PAF.0000000000000542

[25] Yen K, Thali MJ, Aghayev E, Jackowski C, Schweitzer W, Boesch C, et al. Strangulation signs: Initial correlation of MRI, MSCT, and forensic neck findings. J Magn Reson Imaging. 2005;22(4):501–10. 10.1002/jmri.20396. 16142698 10.1002/jmri.20396

[26] Russo MC, Verzeletti A, Piras M, De Ferrari F. Hanging Deaths: A Retrospective Study Regarding 260 Cases. Am J Forensic Med Pathol. 2016;37(3):141–5. 10.1097/paf.0000000000000239. 27281442 10.1097/PAF.0000000000000239

[27] Suicide CSS. Why Are Older Men So Vulnerable? Men Masculinities. 2017;20(1):49–70. 10.1177/1097184x15613832.

[28] Cordner S, Clay FJ, Bassed R, Thomsen AH. Suicidal ligature strangulation: a systematic review of the published literature. Forensic Sci Med Pathol. 2020;16(1):123–33. 10.1007/s12024-019-00187-2. 31773472 10.1007/s12024-019-00187-2

[29] Celo E, Iwaz R, Boucher S, Zabet D, Jousset N. Manual strangulation: When the laryngeal “colossus” gives way. A case report and literature review. Forensic Sci Int Rep. 2024;9:100351. 10.1016/j.fsir.2023.100351.

[30] Godin A, Kremer C, Sauvageau A. Fracture of the cricoid as a potential pointer to homicide. A 6-year retrospective study of neck structures fractures in hanging victims. Am J Forensic Med Pathol. 2012;33(1):4–7. 10.1097/paf.0b013e3181d3dc24. 22442828 10.1097/paf.0b013e3181d3dc24

[31] Malta G, Puntarello M, Midiri M, D’Anna T, Zerbo S, Argo A. Forensic homicidal strangulation in women: Case series and systematic literature review. Forensic Sci Int Synerg. 2025;10:100577. 10.1016/j.fsisyn.2025.100577. 40034148 10.1016/j.fsisyn.2025.100577 PMC11875827

[32] Berend K, Reijnen G. Fatal Manual Strangulation: A Brief Overview. J Forensic Sci Crim Inves. 2023;17:555973. 10.19080/JFSCI.2023.17.555973.

[33] Hoffmann M, Czorlich P, Lehmann W, Spiro AS, Rueger JM, Lefering R. The Impact of Prehospital Intubation With and Without Sedation on Outcome in Trauma Patients With a GCS of 8 or Less. J Neurosurg Anesthesiol. 2017;29(2):161–7. 10.1097/ana.0000000000000275. 26797107 10.1097/ANA.0000000000000275

[34] Fox KR, Franklin JC, Ribeiro JD, Kleiman EM, Bentley KH, Nock MK. Meta-analysis of risk factors for nonsuicidal self-injury. Clin Psychol Rev. 2015;42:156–67. 10.1016/j.cpr.2015.09.002. 26416295 10.1016/j.cpr.2015.09.002 PMC4772426

[35] Cipriano A, Cella S, Cotrufo P. Nonsuicidal Self-injury: A Systematic Review. Front Psychol. 2017;8:1946. 10.3389/fpsyg.2017.01946. 29167651 10.3389/fpsyg.2017.01946 PMC5682335

[36] Kleindienst N, Bohus M, Ludäscher P, Limberger MF, Kuenkele K, Ebner-Priemer UW, et al. Motives for nonsuicidal self-injury among women with borderline personality disorder. J Nerv Ment Dis. 2008;196(3):230–6. 10.1097/NMD.0b013e3181663026. 18340259 10.1097/NMD.0b013e3181663026

[37] Manninen A‑L, Isokangas J‑M, Karttunen A, Siniluoto T, Nieminen MT. A Comparison of Radiation Exposure between Diagnostic CTA and DSA Examinations of Cerebral and Cervicocerebral Vessels. Am J Neuroradiol. 2012;33(11):2038–42. 10.3174/ajnr.A3123. 22700752 10.3174/ajnr.A3123 PMC7965573

[38] Bos D, Guberina N, Zensen S, Opitz M, Forsting M, Wetter A. Radiation Exposure in Computed Tomography. Dtsch Ärztebl Int. 2023;120(9):135–41. 10.3238/arztebl.m2022.0395. 36633449 10.3238/arztebl.m2022.0395 PMC10198168

[39] Hauptmann M, Byrnes G, Cardis E, Bernier MO, Blettner M, Dabin J, et al. Brain cancer after radiation exposure from CT examinations of children and young adults: results from the EPI-CT cohort study. Lancet Oncol. 2023;24(1):45–53. 10.1016/s1470-2045(22)00655-6. 36493793 10.1016/S1470-2045(22)00655-6

[40] Ruder TD, Gonzenbach A, Heimer J, Arneberg L, Klukowska-Rötzler J, Blunier S, et al. Imaging of alert patients after non-self-inflicted strangulation: MRI is superior to CT. Eur Radiol. 2024;34(6):3813–22. 10.1007/s00330-023-10354-3. 37953368 10.1007/s00330-023-10354-3 PMC11166758

[41] Mozayan M, Sexton C. Imaging of carotid artery dissection. J Community Hosp Intern Med Perspect. 2012; 10.3402/jchimp.v2i2.18645. 23882367 10.3402/jchimp.v2i2.18645 PMC3714059

[42] Heldner MR, Nedelcheva M, Yan X, Slotboom J, Mathier E, Hulliger J, et al. Dynamic changes of intramural hematoma in patients with acute spontaneous internal carotid artery dissection. Int J Stroke. 2015;10(6):887–92. 10.1111/ijs.12553. 26121371 10.1111/ijs.12553

[43] Liu Q, Huang J, Degnan AJ, Chen S, Gillard JH, Teng Z, et al. Comparison of high-resolution MRI with CT angiography and digital subtraction angiography for the evaluation of middle cerebral artery atherosclerotic steno-occlusive disease. Int J Cardiovasc Imaging. 2013;29(7):1491–8. 10.1007/s10554-013-0237-3. 23686460 10.1007/s10554-013-0237-3

[44] Saba L, Yuan C, Hatsukami TS, Balu N, Qiao Y, DeMarco JK, et al. Carotid Artery Wall Imaging: Perspective and Guidelines from the ASNR Vessel Wall Imaging Study Group and Expert Consensus Recommendations of the American Society of Neuroradiology. AJNR Am J Neuroradiol. 2018;39(2):E9–e31. 10.3174/ajnr.A5488. 29326139 10.3174/ajnr.A5488 PMC7410574

[45] Debette S, Compter A, Labeyrie MA, Uyttenboogaart M, Metso TM, Majersik JJ, et al. Epidemiology, pathophysiology, diagnosis, and management of intracranial artery dissection. Lancet Neurol. 2015;14(6):640–54. 10.1016/s1474-4422(15)00009-5. 25987283 10.1016/S1474-4422(15)00009-5

[46] Li X, Su F, Yuan Q, Chen Y, Liu CY, Fan Y. Advances in differential diagnosis of cerebrovascular diseases in magnetic resonance imaging: a narrative review. Quant Imaging Med Surg. 2023;13(4):2712–34. 10.21037/qims-22-750. 37064346 10.21037/qims-22-750 PMC10102759

[47] Kim JJ, Gean AD. Imaging for the Diagnosis and Management of Traumatic Brain Injury. Neurother. 2011;8(1):39–53. 10.1007/s13311-010-0003-3. 10.1007/s13311-010-0003-3 PMC3026928 21274684

